# Low Prevalence of Folic Acid Supplementation during Pregnancy: A Multicenter Study in Vietnam

**DOI:** 10.3390/nu11102347

**Published:** 2019-10-02

**Authors:** Anh Vo Van Ha, Yun Zhao, Colin W. Binns, Ngoc Minh Pham, Cong Luat Nguyen, Phung Thi Hoang Nguyen, Tan Khac Chu, Andy H. Lee

**Affiliations:** 1 School of Public Health, Curtin University, Perth WA 6845, Australia; vovananh.ha@postgrad.curtin.edu.au (A.V.V.H.); C.Binns@curtin.edu.au (C.W.B.); minh.pn@tnu.edu.vn (N.M.P.); congluat@gmail.com (C.L.N.); nthphungytcc@ump.edu.vn (P.T.H.N.); cktan@hpmu.edu.vn (T.K.C.); Andy.Lee@curtin.edu.au (A.H.L.); 2 Faculty of Public Health, Pham Ngoc Thach University of Medicine, Ho Chi Minh City 700000, Vietnam; 3 Faculty of Public Health, Thai Nguyen University of Medicine and Pharmacy, Thai Nguyen 250000, Vietnam; 4 National Institute of Hygiene and Epidemiology, Hanoi 100000, Vietnam; 5 Faculty of Public Health, University of Medicine and Pharmacy at Ho Chi Minh City, Ho Chi Minh City 700000, Vietnam; 6 Faculty of Public Health, Hai Phong University of Medicine and Pharmacy, Hai Phong 180000, Vietnam

**Keywords:** folic acid, folate, pregnancy, dietary supplement, Vietnam

## Abstract

Periconceptional folic acid (FA) supplementation is recommended to prevent neural tube defects (NTDs), but little information is known about its use in Vietnam. It is important that FA supplements start to be taken when planning a pregnancy and continued through the first trimester to prevent NTDs, as the neural tube closes in the first month of pregnancy. However, FA supplementation in Vietnam is usually recommended to commence from the first antenatal visit, which is usually at 16 weeks, and very few women take FA before their first visit. This multicenter study aimed to determine the prevalence of FA supplement use and associated maternal characteristics in Vietnam. FA supplementation was assessed in 2030 singleton pregnant women between 2015 and 2016. In total, 654 (32.2%) women reported taking either supplements containing FA alone or multivitamins containing FA, and 505 (24.9%) reported correctly taking supplements containing FA alone. Women who were aged 30 years or over, had low education levels, had formal employment, and whose current pregnancy was first or unplanned were less likely to supplement with FA. Education programs are needed to encourage FA supplementation when contemplating pregnancy.

## 1. Introduction

Folate has a vital role in periods of rapid cell division and growth, especially during pregnancy [1]. It is difficult to measure folate intake from food because it exists in multiple forms of polyglutamates, which have varying levels of bioactivity and can easily be destroyed by sunlight, cooking and storage processes [2]. Folic acid (FA) is the synthetic form of folate that is used in supplements and food fortification. FA only contains the monoglutamate form, which is more biologically active than polyglutamates and is more efficient for preventing adverse health effects due to folate deficiency [2]. 

Folate deficiency is one of the most common micronutrient deficiencies during pregnancy, as the demand for folate is increased due to fetal growth and development. This can occur if the mother’s dietary intake of folate is inadequate during pregnancy [3,4,5,6]. The cells’ ability to methylate important compounds, including DNA, proteins and lipids, may be compromised by folate deficiency, leading to impaired cellular function [7]. The impairment of DNA synthesis or methylation reactions can adversely affect genomic responses (e.g., impaired gene expression, genomic instability and DNA damage), possibly preventing the proper closure of the neural tube [8]. Intervention studies have demonstrated the protective effects of FA supplementation against some types of neural tube defects (NTDs) [9,10,11] and congenital heart defects [12,13]. In order to prevent NTDs, the World Health Organization (WHO) recommends periconceptional folic acid supplementation, i.e., that all women take a daily supplement of 400 μg FA from the moment they plan to conceive until 12 weeks of gestation [14]. Following confirmation by the Medical Research Council Vitamin Study in 1991, most Western countries have recommended FA supplementation [15,16,17]. In Vietnam, the latest national guidelines on nutrition for pregnant women (in accordance with the Decision 776/2017-QD-BYT) were issued in 2017, which recommend supplementation with a daily dose of 400 μg FA during pregnancy. However, it is recommended that health professionals start the prescription of FA supplements to pregnant women at their first antenatal visit [18], which, on average, is at four months of pregnancy [19], i.e., later than the date recommended by the WHO [14].

Congenital anomalies that can be prevented by FA supplementation continue to be one of the top three causes of neonatal mortality in Vietnam [20]. The prevalence of anencephaly (a type of NTD) was estimated at 3.6 per 10,000 live births in Vietnam in 2010, which is higher than the corresponding data in the USA (1.9–2.3 per 10,000 live births during 1995–2011) and Thailand (0.8 per 10,000 live births during 2001–2012) [21,22]. The prevalence of NTDs declined slightly from 62.1 per 100,000 live births to 61.7 per 100,000 live births during the past 10 years (from 2007 to 2017), but was still higher than the corresponding rates in Singapore (12.4 per 100,000 live births) and Cambodia (45.3 per 100,000 live births) in 2017 [23]. The awareness of the importance of FA prior to conception and during early pregnancy is low, and little information is available on the coverage of supplementation with FA during this period [24,25,26,27]. Studies that reported the coverage of FA supplementation were not able to assess the frequency and duration of supplementation. In addition, no health promotion programs have been focused on FA supplementation to prevent NTDs at national and community level in Vietnam [28].

Previous studies have shown the relationships between some maternal characteristics and FA supplementation; however, to date, very few studies have been conducted in Asian countries [29,30,31,32]. For examples, the authors noted that maternal age, parity, and a planned pregnancy are associated with FA supplementation [30,32]. A randomized controlled trial in Vietnam reported several factors associated with the use of prenatal micronutrient supplements by pregnant women [26]. However, the study was conducted in only one rural province. Information on FA supplementation following the WHO recommendations in large-scale studies of Vietnamese pregnant women is lacking. 

The objective of this multicenter study was to determine the prevalence of correct FA supplementation among Vietnamese pregnant women and to ascertain the maternal characteristics associated with FA use. The findings of this study are important to ensure that FA supplementation is conducted in accordance with the WHO recommendations and to update or develop national nutrition strategies on promoting FA supplementation during pregnancy, and thereby to reduce the burden of NTDs in Vietnam. 

## 2. Materials and Methods

Details of the study design and recruitment procedure have been described elsewhere [33]. This study collected data at the first (baseline) interview (undertaken between August 2015 and July 2016) from a multicenter cohort study conducted in Hanoi, Hai Phong and Ho Chi Minh City. Among the 2248 eligible pregnant women recruited from six participating hospitals, 218 (9.7%) women declined to participate due to time constraints or planned relocation after delivery, and 2030 women (a response rate of 90.3%) agreed to participate and gave their informed consent. The inclusion criteria were as follows: (1) permanent residents in the study locations; (2) ≥18 years of age; (3) singleton pregnancy; and (4) able to read the information sheet and sign the consent form. The latter condition was not invoked, because there is almost universal literacy in Vietnam. Women with serious pre-existing health conditions such as cancer or ischemic heart disease were excluded after consultation with their medical doctors. This study was approved by the Human Research Ethics Committees of Curtin University, Australia (HR32/2015) and Hai Phong University of Medicine and Pharmacy, Vietnam (No. 05/HPUMPRB/2015). Confidentiality was assured and written informed consent was obtained from all participants. 

Trained interviewers collected information on dietary supplementation via a validated Food Frequency Questionnaire at the baseline interview [4,34]. Upon receiving a positive response to the question “Have you ever taken FA/multivitamin supplements during this pregnancy?”, further consumption details were solicited in terms of frequency (times per day/week/month), quantity (number of tablets taken each time) and duration (number of months/weeks/days taken during pregnancy) of supplementation. The information was cross-checked against their pregnancy health books and medical records whenever possible and subsequently reconfirmed with the participants if any discrepancy was found. 

Given that the multivitamin supplements available in Vietnam contain FA, the first binary outcome variable of our study was defined as the correct use of either supplements containing FA alone or multivitamins containing FA (1 = correct use, 0 = incorrect use). The second binary outcome variable was defined as the correct use of supplements containing FA alone (1 = correct use, 0 = incorrect use). We did not intentionally assess the amount of FA taken, though prenatal supplements provided to pregnant women are required to provide at least 600 µg of FA daily (Circular No.43/2014/TT-BYT) [35]. In the Vietnamese market, common commercial prenatal supplements contain approximately 400 µg FA [36,37]. Therefore, for both outcomes, “correct use” in this study was defined as the daily use of a supplement containing 400 µg (or more) FA and commencement of supplementation from the time when the pregnancy was planned until 12 weeks of gestation (periconceptional period), following the WHO recommendations [14]. The “incorrect use” group included participants who never consumed FA from supplements, those who did not take the tablets on a daily basis, and those who took the tablets for a shorter duration than that recommended by the WHO. 

Information on maternal characteristics was also obtained during the baseline interview, including age (<25, 25–29, and ≥30 years), formal employment (yes = office-based staff, service, sales or manufacturing workers and technicians; no = housewives, freelancers, unskilled farmers or planters, unemployed), education level (secondary school or lower; high school; college or university), parity (nulliparous; multiparous), pre-pregnancy weight, and whether or not the current pregnancy was planned (yes; no). Maternal height was measured using a stadiometer to the nearest 1 mm. The pre-pregnancy body mass index (BMI) value was then calculated using the self-reported pre-pregnancy weight and height, and classified according to the WHO criteria for Asian populations [38] as underweight (<18.5 kg/m^2^), normal (18.5–22.9 kg/m^2^), or overweight/obese (≥23 kg/m^2^).

Descriptive statistics were used to summarize the maternal characteristics and estimate the prevalence of correctly taking (1) either supplements containing FA alone or multivitamins containing FA, and (2) supplements containing FA alone at a daily dose of 400 µg (or more). We then performed univariate (for each independent variable) and multivariable logistic regression analyses to separately determine the associations between the maternal characteristics and either of the two outcome variables. All independent variables considered in the full regression models (maternal age, education level, formal employment, pre-pregnancy BMI, parity, and planned pregnancy) were based on the available information and with reference to the literature as plausible factors affecting FA use [39,40,41,42,43,44,45]. The results are presented as crude (unadjusted), and adjusted odds ratios (ORs) with 95% confidence intervals (CIs). All statistical analyses were performed using Stata version 15.1 [46].

## 3. Results

The mean age of the 2030 women included in this study was 27.6 years (Standard Deviation (SD) = 5.3), and over one-third (35.6%) of women only attended secondary school or had a lower level of education. In addition, a total of 1393 women (68.6%) were formally employed. For 789 women (38.9%), it was their first pregnancy, and 601 women (29.7%) stated that they had not planned their current pregnancy. Before pregnancy, the prevalence of underweight and overweight/obese was 26.1% and 12.7%, respectively. 

There were 522 (25.7%) Vietnamese pregnant women who took supplements containing FA alone, while 675 (33.2%) took either supplements containing FA alone or multivitamins containing FA when the pregnancy was planned until 12 weeks of gestation. Overall, 654 (32.2%) pregnant women reported correct use of either supplements containing FA alone or multivitamins containing FA, while 505 (24.9%) reported correct use of supplements containing FA alone during the periconceptional period (Table 1). Notably, 50 mothers (2.5%) never took any such dietary supplements from the time when the pregnancy was planned until 12 weeks of gestation. 

Table 2 and Table 3 show the results of the univariate and multivariable logistic regression analyses. Inverse associations were evident between both correct supplement use outcomes and maternal age ≥30 years, secondary school, or a lower level of education, and nulliparous or unplanned pregnancy. Attending high school and having formal employment were only significantly associated with reduced odds of correctly taking supplements containing FA alone or multivitamins containing FA.

## 4. Discussion

This is the first observational study to report the prevalence of correct FA supplementation as recommended by the WHO in Vietnamese women. It also provides an insight into the association between maternal characteristics and the correct supplemental intake of periconceptional FA as recommended by the WHO. Despite the WHO recommendations that women take a daily FA supplement from the time when the pregnancy was planned until 12 weeks of gestation, only 32.2% of women (*n* = 654) reported taking either supplements containing FA alone or multivitamins containing FA, and one in four women (24.9%, *n* = 505) reported taking supplements containing FA alone at a daily dose of 400 µg (or more), and started when the pregnancy was planned and continued until 12 weeks of gestation. These findings suggest that FA supplementation in accordance with the WHO recommendations remains a major challenge in Vietnam. Our findings on the low prevalence of correct FA supplementation (or high prevalence of incorrect FA supplementation) suggests that the Vietnam national guidelines on nutrition for pregnant women need to be adjusted according to the WHO recommendations, with an emphasis on correct timing (from when planning to conceive), correct amount (400 μg daily), and sufficient duration (12 weeks of gestation) of FA supplement use.

The observed prevalence of correct FA supplementation was lower than the results reported in China (67.7%), but higher than those reported in some high-income countries in Asia, such as Japan (20.5%) and Korea (10.3%) [30,32,44]. Compared with some Western countries, the prevalence of correct FA supplementation found in our study was lower than that found in Ireland (43.9%) between 2009 and 2013 but was comparable to corresponding data in New Zealand collected in 2010 (33.3%) [42,47]. The differences in FA supplementation prevalence among countries might result from variations in policy promotion and practical implementations. 

Our study found that an education level of secondary school or lower education was associated with lower odds of correct FA supplementation. This finding is consistent with other studies in the literature, which reported that women with higher education levels were more likely to correctly take FA-containing supplements than those with low education level [31,39,40,48,49], and were at higher risk for severe maternal/infant outcomes [50]. Mothers with lower education levels may lack awareness, knowledge, and confidence regarding the WHO recommendations for FA supplementation. They might be unaware of the benefits of FA to the healthy fetal growth and development, and the potential susceptibility to, and severity of NTDs caused by folate deficiency [27]. Our study also showed that Vietnamese women aged 30 years or over were less likely to comply with the WHO recommendation, contrary to previous observations in Japan, the Netherlands, and Norway [32,39,47]. Reasons for the discrepancy may include a lack of awareness of older Vietnamese women concerning the role of FA in reducing the risk of NTDs, and their belief that dietary intake alone is sufficient to prevent the defect.

Studies in developed countries have shown that unskilled or semi-skilled manual workers or those without paid employment are less likely to consume FA from supplements [41,47,51]. Our study showed that Vietnamese women in formal employment were associated with lower odds of correctly taking either supplements containing FA alone or multivitamins containing FA as recommended by the WHO. In Vietnam, married women are expected to work, take care of children and other family (and family-in-law) members, and do housework [52]. It is thus plausible that they are more likely to forget or skip prescribed supplementation during pregnancy if they have a formal employment that is often full-time with a fixed work schedule [27]. The group with formal employment may be difficult to reach in community-based health promotion programs due to their tight schedules. Our findings suggest that workplace-based campaigns might be a more appropriate way to access to women in these latter groups. 

If the current pregnancy was the first pregnancy or was unplanned, women might not be aware of the need for FA supplementation during pregnancy. Most Vietnamese women began supplementation only after becoming pregnant [27]. Likewise, our study found that women undergoing their first pregnancy and those with an unplanned pregnancy were associated with lower likelihoods of correctly taking FA-containing supplements. In view of the declining fertility rate in Vietnam from 3.6 to 2.0 births per woman during 1990–2017 [53], it is important to give greater attention to Vietnamese primigravida as well as women who have just married regarding the correct use of FA-containing supplements, because they may account for a large number of pregnancies in Vietnam in the near future. 

Antenatal care services play an important role in promoting and implementing the correct use of FA-containing supplements. However, in Vietnam, only approximately 4% of women aged 15–49 years with a live birth within the past two years attended the first antenatal care visit during the first trimester [19] and very few mothers attend these services until the second trimester [54]. Additionally, health professionals may not be fully aware of FA recommendations in Vietnam [55]. Both a lack of antenatal care attendance and lack of full awareness in health professionals contribute to the low prevalence of correct FA supplementation during the periconceptional period. A randomized controlled trial on prenatal micronutrient supplements indicated a positive relationship between the number of community health worker visits and adherence to prenatal supplementation in Vietnam [26]. These findings suggest that the support of community health services may improve the prevalence of correct FA supplementation recommended by the WHO.

Due to the absence of a national birth defect surveillance system, there is a lack of complete information on NTDs that could be used to advocate for interventions on FA supplementation [56,57]. The Vietnam National Nutrition Strategy for 2011–2020 does not mention the promotion of FA supplementation [28]. As a result, Vietnamese women may not be aware of the critical role of FA during pregnancy and may therefore not adhere to FA prescriptions [27]. Indeed, the recent national guidelines on nutrition for pregnant women recommend FA supplementation; however, the supplementation is only recommended to commence from the first antenatal care visit [18], which is commonly at approximately 16 weeks of pregnancy in Vietnam [19]. Given that women do not typically plan their pregnancy and attend antenatal care later in gestation, women of reproductive age should be targeted in programs promoting FA supplementation. In addition, health communication through multiple media platforms may be tailored to motivate the imperative of FA supplementation and improve the utilization of antenatal health care services, particularly in women who were found to be less likely to supplement FA correctly in this study.

Our study was strengthened by its multicenter cohort design, relatively large representative sample size and high response rate (90%). Another notable strength is that we examined the details of the use of FA in pregnant Vietnamese women using two separate outcome variables, namely either the correct use of either supplements containing FA alone or multivitamins containing FA, and the correct use of supplements containing FA alone, in both bivariate and multivariable logistic regression analyses. There are several limitations to be considered when interpreting the results of this study. Regional effect was not assessed, because the sample size of one region was relatively small to enable a valid comparison between study regions. The present study recruited pregnant women who sought prenatal care at public hospitals but not those attending private health clinics. Little information is available concerning the contribution of private health facilities or private practitioners to reproductive health in Vietnam [54]. The brands of supplements were not assessed in detail, but we listed the most common brands during the interview. Finally, we did not evaluate FA intake from fortified food. Voluntary wheat flour fortification with FA has been promoted since 2003, but few products are available on sale. Rice is the staple food in Vietnam, yet its fortification remains difficult to implement in practice. 

## 5. Conclusions

The prevalence of correct FA supplementation during pregnancy was found to be low in Vietnam. Less than one-third of women reported taking either supplements containing FA alone or multivitamins containing FA, in accordance with the WHO recommendations. Based on our findings, greater attention should be focused on mothers who are aged thirty years or over, have only attained an education level of secondary school or lower, are formally employed, and having their first pregnancy, together with promote pregnancy planning in couples. The results suggest that more campaigns promoting FA supplementation might help raise the consciousness and awareness of the importance of FA, especially in these vulnerable subgroups of women. In order to ensure the correctness of FA use among all Vietnamese women, the timing of FA supplementation commencement in preventing NTDs should be emphasized to a greater extent as part of these health campaigns.

## Figures and Tables

**Table 1:** Periconceptional supplementation with folic acid (FA), Vietnam 2015–2016 (*n* = 2030).

Intakes	Either Supplements Containing FA Alone or Multivitamins Containing FA			Supplements Containing FA Alone		
	*n (%)*			*n (%)*		
Frequency	Daily	Non-daily	Did not use	Daily	Non-dail	Did not use
	1912 (94.2)	68 (3.3)	50 (2.5)	1702 (83.8)	67 (3.3)	261 (12.9)
Duration (when the pregnancy was planned)	Until 12 weeks of gestation	<12 weeks of gestation	Did not use	Until 12 weeks of gestation	<12 weeks of gestation	Did not use
	675 (33.2)	1305 (64.3)	50 (2.5)	522 (25.7)	1247 (61.4)	261 (12.9)
Correct use of FA	Correct use ^1^	Incorrect use ^2^		Correct use ^1^	Incorrect use ^2^	
	654 (32.2)	1376 (67.8)		505 (24.9)	1525 (75.1)	

FA = folic acid. ^1^ Includes daily users of supplements containing 400 µg (or more) FA who started supplementation when the pregnancy was planned and continued until 12 weeks of gestation. ^2^ Includes participants who never consumed FA from the supplements, who did not take the tablets on a daily basis, and who took the tablets for a shorter duration than that recommended by the WHO [14].

**Table 2:** Maternal characteristics associated with the correct use of supplements containing folic acid alone during the periconceptional period in Vietnam, 2015–2016 (*n* = 2030).

Characteristic	Correct Use ^4^ (*n* = 505)	OR (95% CI) ^5^	*p*	OR (95% CI) ^1,6^	*p*
	*n (%)*				
Maternal age (years)					
<25	181 (35.8)	1		1	
25–29	188 (37.2)	0.90 (0.71, 1.14)	0.400	0.82 (0.63, 1.06)	0.128
≥30	136 (27.0)	0.63 (0.49, 0.82)	<0.001	0.57 (0.42, 0.76)	<0.001
Education level					
Secondary school or lower	133 (26.3)	0.60 (0.47, 0.77)	<0.001	0.61 (0.47, 0.79)	<0.001
High school	159 (31.5)	1.16 (0.91, 1.48)	0.226	1.11 (0.87, 1.43)	0.402
College or university	213 (42.2)	1		1	
Formal employment					
No	466 (30.6)	1		1	
Yes	1059 (69.4)	0.86 (0.69, 1.06)	0.166	0.81 (0.65, 1.02)	0.072
Pre-pregnancy BMI (kg/m^2^) ^2^					
Underweight (<18)	139 (27.5)	1.05 (0.84, 1.33)	0.654	1.00 (0.79, 1.27)	0.984
Normal (18–22.9)	314 (62.2)	1		1	
Overweight/obese (≥23)	52 (10.3)	0.75 (0.54, 1.04)	0.083	0.87 (0.62, 1.22)	0.420
Parity					
Nulliparous	182 (36.0)	0.85 (0.69, 1.05)	0.133	0.62 (0.48, 0.78)	<0.001
Multiparous	323 (64.0)	1		1	
Planned pregnancy ^3^					
No	113 (22.4)	0.61 (0.48, 0.77)	<0.001	0.60 (0.47, 0.76)	<0.001
Yes	392 (77.6)	1		1	

OR = Odds ratio. CI = confidence interval. BMI = body mass index. ^1^ From the full logistic regression model, which included maternal age, education level, formal employment, pre-pregnancy BMI, parity, and planned pregnancy. ^2^ Based on the cut-off for the Asian population [34]. ^3^ Missing data presents. ^4^ Includes the daily users of supplements containing 400 µg (or more) folic acid who started supplementation when the pregnancy was planned and continued until 12 weeks of gestation [14]. ^5^ Crude odds ratio. ^6^ Adjusted odds ratio.

**Table 3:** Maternal characteristics associated with the correct use of either supplements containing folic acid alone or multivitamins containing folic acid during the periconceptional period in Vietnam, 2015–2016 (*n* = 2030).

Characteristic	Correct Use ^4^ (*n* = 654)	OR (95% CI) ^5^	*p*	OR (95% CI) ^1,6^	*p*
	*n (%)*				
Maternal age (years)					
<25	222 (33.9)	1		1	
25–29	259 (39.6)	1.07 (0.85, 1.33)	0.572	0.96 (0.75, 1.22)	0.720
≥30	173 (26.5)	0.64 (0.50, 0.81)	<0.001	0.63 (0.48, 0.83)	0.001
Education level					
Secondary school or lower	155 (23.7)	0.40 (0.32, 0.51)	<0.001	0.41 (0.32, 0.52)	<0.001
High school	183 (28.0)	0.79 (0.63, 0.99)	0.045	0.77 (0.61, 0.98)	0.032
College or university	316 (48.3)	1		1	
Formal employment					
No	217 (33.2)	1		1	
Yes	437 (66.8)	0.88 (0.72, 1.08)	0.228	0.76 (0.62, 0.94)	0.012
Pre-pregnancy BMI (kg/m^2^) ^2^					
Underweight (<18)	177 (27.1)	1.00 (0.81, 1.24)	0.997	0.94 (0.76, 1.18)	0.619
Normal (18–22.9)	416 (63.6)	1		1	
Overweight/obese (≥23)	61 (9.3)	0.62 (0.45, 0.84)	0.002	0.76 (0.55, 1.05)	0.093
Parity					
Nulliparous	250 (38.2)	0.96 (0.79, 1.16)	0.683	0.67 (0.54, 0.84)	0.001
Multiparous	404 (61.8)	1		1	
Planned pregnancy ^3^					
No	149 (22.8)	0.60 (0.49, 0.75)	<0.001	0.62 (0.50, 0.77)	<0.001
Yes	505 (77.2)	1		1	

OR = Odds ratio. CI = confidence interval. BMI = body mass index. ^1^ From the full logistic regression analysis, which included maternal age, education level, formal employment, pre-pregnancy BMI, parity, and planned pregnancy. ^2^ Based on the cut-off for the Asian population [38]. ^3^ Missing data presents. ^4^ Includes the daily users of supplements containing 400 µg (or more) folic acid who started supplementation when the pregnancy was planned and continued until 12 weeks of gestation [14]. ^5^ Crude odds ratio. ^6^ Adjusted odds ratio.
